# Acute Ventricular Dysfunction After Doxorubicin-Based Induction Therapy for Pediatric Acute Lymphoblastic Leukemia

**DOI:** 10.7759/cureus.75720

**Published:** 2024-12-14

**Authors:** Rajkumar Kundavaram, Amber Kumar, Sushma Konnepati, Yogendra S Yadav, Narendra K Chaudhary, Shikha Malik, Priya Gogia

**Affiliations:** 1 Pediatrics, All India Institute of Medical Sciences, Bhopal, IND; 2 General Medicine, All India Institute of Medical Sciences, Bhopal, IND

**Keywords:** acute leukemia, cardiotoxicity, doxorubicin, mpi, tdi

## Abstract

Background

Doxorubicin is an important drug used in the treatment of children with acute leukemia, and cardiotoxicity is the most serious complication due to its use. The cardiac dysfunction due to doxorubicin can be acute, early, or late. Echocardiography is a non-invasive tool and can be employed to detect clinical and subclinical cardiac dysfunction and plan treatment strategies accordingly.

Materials and methods

Twenty-eight children with acute lymphoblastic leukemia were enrolled. Echocardiography was done at baseline and 72 h after induction dose of doxorubicin. Conventional and tissue Doppler imaging parameters were obtained and compared.

Results

After the induction dose of doxorubicin, both ventricles developed systolic and diastolic dysfunction. Tricuspid annular plane systolic excursion significantly decreased after doxorubicin (20.0±4.95 mm vs. 19.40±4.90 mm). Right ventricular myocardial performance index and isovolumetric relaxation times increased after doxorubicin (0.38±0.08 vs. 0.41±0.08 and 41.4±1.8 ms vs. 43.1±12.6 ms, p<0.05, respectively). Tricuspid E velocity decreased (62.3±8.35 cm/s vs. 60.1±7.34 cm/s, p<0.01) as well as tricuspid E/A ratio after doxorubicin (1.54±0.26 vs. 1.40±0.23, p<0.01). The left ventricular fractional shortening and ejection fraction decreased after doxorubicin (32.1±2.26% vs. 31.4±2.27% and 64.60±4.69% vs. 63.10±4.63%, respectively). Left ventricular myocardial performance index and isovolumetric relaxation times were increased after doxorubicin (0.44±0.05 vs. 0.46±0.06 and 58.6±8.75 ms vs. 60.3±10.1 ms, respectively). Mitral E velocity is reduced (85.6±11.3 cm/s vs. 83±11.9 cm/s) and tricuspid E/A ratio is also reduced after doxorubicin (1.78±0.43 vs. 1.63±0.39).

Conclusion

Both systolic and diastolic dysfunctions are seen after doxorubicin. Echocardiography should be employed for early diagnosis of clinical and subclinical cardiac dysfunction and timely initiation of management to prevent progression.

## Introduction

Advances in diagnostics and the availability of novel therapeutic agents have brought tremendous improvement in cancer survival rates in children. However, the burden of treatment-related side effects is also increasing. Anthracyclines (doxorubicin, daunorubicin, epirubicin) are by far the most commonly used chemotherapeutic agents in children [1] and cardiotoxicity remains one of the major adverse effects following treatment with these drugs [2]. Childhood cancer survivors had tenfold cardiac-related mortality as compared with healthy controls [3]. As per Cardinale et al., anthracycline-induced cardiotoxicities can be categorized as per the time of onset described as follows: acute effects occur following a single dose and up to two weeks of administration, early effects are the most common form and develop within a year, and late-onset chronic effects occur beyond one year after the end of chemotherapy [4]. While there are many studies reporting the early and late cardiotoxic effects, studies on acute effects are sparse in children.

## Materials and methods

This is a cross-sectional prospective study and included 28 children who are newly diagnosed with acute lymphoblastic leukemia at the Department of Pediatrics, All India Institute of Medical Sciences, Bhopal, India. Prior to enrolment, written informed consent was obtained from the parents or legal guardians. Assent was obtained when required. Institutional ethics committee approval was obtained before enrolling patients (ref no: IHEC/SR/2024/116). History taking, general examination, laboratory evaluation, and cardiac examinations were done and the treatment protocols were initiated as per standard guidelines. These patients received 25 mg/m^2^ of doxorubicin on day eight of the induction phase.

Echocardiography image acquisition

Single pediatric cardiologist has performed transthoracic echocardiography of all patients before and 72 h after the induction dose of doxorubicin. The patients were examined in supine and left lateral positions with the Philips, Epiq7 system (Amsterdam, Netherlands: Philips Ultrasound LLC). The frequency of the probe used was 3 or 5 MHz as per the age of patient. For timing cardiac cycle events, ECG gating was performed with the beginning of the QRS complex as the reference point. The pediatric cardiologist was blinded for patient status (before or after chemotherapy).

Conventional Echo-Doppler Parameters

The conventional measures included two-dimensional (2D), motion mode (M-mode), pulsed, and continuous wave Doppler velocity (PW and CW Doppler) of the cardiac valves. Measures of M-mode were the following left ventricular (LV) dimensions: left ventricular internal diameter in diastole (LVIDd) and left ventricular internal diameter in systole (LVIDs), fractional shortening (FS), and ejection fraction (EF). For the right ventricle (RV), tricuspid annular plane systolic excursion (TAPSE) was measured. Doppler parameters obtained included velocities and ratio (peak E, peak A, E/A ratio) for both the tricuspid and mitral valves.

Tissue Doppler Imaging

Tissue Doppler imaging (TDI) was used to obtain E’, E/E’, and isovolumetric relaxation time (IVRT). Myocardial performance index (MPI or Tei index) was obtained for both ventricles using pulsed wave tissue Doppler imaging (PW-TDI): at the tricuspid annulus for RV and the lateral mitral annulus for LV. The parameters like isovolumetric contraction time (IVCT) and ejection time (ET) were also obtained. All the intervals’ measurements were obtained within one cardiac cycle. The following formula was used for calculating the Tei index: MPI = (IVCT+IVRT)/ET [5]. Since breath holding was not feasible in these young children, we recorded three cardiac cycles, and the times and velocities were averaged to reduce the effect of respiratory variation on tissue velocities. Appropriate care was taken to keep the ultrasound beam angle below 15°, and temporal resolution was enhanced by maintaining a frame rate (FPS) above 200 [6].

Statistical Analysis

Statistical analysis was done using Jamovi statistical software (version 2.4.12; Sydney, Australia: JASP project). The data were assessed for normal distribution using Kolmogorov-Smirnov test. Mean±SD was used when the parameters were normally distributed and percentages were used for categorical data. Student's t-test was used to compare groups with normally distributed data and Wilcoxon test was used to compare groups with non-normally distributed data. Pearson’s and Spearman’s coefficients were utilized to look for correlations. Statistical significance was considered with p-values <0.05.

## Results

A total of 28 patients were enrolled, including 18 males (64%). The median age was 5.0 years (IQR: 3.75-13.3). Table 1 shows the patient characteristics. The median hemoglobin concentration was 8.55 g/dL (IQR: 7.25-9.53), and the median platelet count was 0.25×10³/mm³ (IQR: 0.14-0.46×10³). The laboratory parameters are presented in Table 2.

**Table 1:** Demographic profile of study participants (n=28). BSA: body surface area; B ALL: B-cell acute lymphoblastic leukemia; T ALL: T-cell acute lymphoblastic leukemia

Parameter	Value
Male, n (%)	18 (64)
Female, n (%)	10 (36)
Age (years) median (IQR)	5.0 (3.75-13.3)
Weight (kg) median (IQR)	15.6 (12.4-40.3)
Height (cm) median (IQR)	107 (95.8-149)
BSA (m^2^) median (IQR)	0.66 (0.55-1.29)
B ALL, n (%)	23 (82)
T ALL, n (%)	5 (18)

**Table 2:** Laboratory parameters of study participants.

Parameter	Median (IQR)
Hemoglobin (g/dL)	8.55 (7.25-9.53)
Platelets (x10^5^/mm^3^)	0.25 (0.14-0.46)
Total leucocyte count (x10^3^/mm^3^)	5.14 (1.5-9.7)
Absolute neutrophil count (x10^3^/mm^3^)	0.51 (0.2-3.1)
Absolute lymphocyte count (x10^3^/mm^3^)	2.06 (0.95-3.92)
Potassium (mEq/dL)	4.05 (3.59-4.40)
Calcium (mEq/dL)	8.57 (7.99-9.14)
Phosphate (mg/dL)	3.83 (3.14-4.96)
Uric acid (mg/dL)	1.83 (1.23-3.38)

Right ventricular functions before and after doxorubicin

Isovolumetric contraction time (IVCT) and tricuspid E’/E did not show significant change after doxorubicin. However, other parameters of systolic and diastolic dysfunction were significantly affected: tricuspid annular plane systolic excursion (TAPSE) significantly decreased after doxorubicin (20.0±4.95 mm vs. 19.40±4.90 mm, p<0.01) (Table 3).

**Table 3:** Right ventricular functions before and after doxorubicin. TAPSE: tricuspid annular plane systolic excursion; RV IVCT: right ventricular isovolumetric contraction time; RV IVRT: right ventricular isovolumetric relaxation time; RV MPI: right ventricular myocardial performance index

Parameter	Before (n=28)	After (n=28)	p-Value	t-Statistic
TAPSE (mm)	20.0 (4.95)	19.4 (4.90)	<0.01	12.18
RV IVCT (ms)	38.9 (10.9)	39.8 (10.6)	0.001	-3.35
RV IVRT (ms)	41.4 (11.8)	43.1 (12.6)	<0.01	-4.69
RV MPI (ms)	0.380 (0.08)	0.413 (0.08)	<0.01	-7.11
Tricuspid E velocity (cm/s)	62.3 (8.35)	60.1 (7.34)	<0.01	6.97
Tricuspid A velocity (cm/s)	41.4 (8.28)	43.7 (8.31)	<0.01	-7.36
Tricuspid E/A	1.54 (0.26)	1.40 (0.23)	<0.01	9.50
Tricuspid E’ velocity (cm/s)	15.0 (3.37)	14.0 (2.84)	<0.01	5.98
Tricuspid E/E’	4.25 (0.57)	4.37 (0.59)	1.000	-4.80

Myocardial performance index (MPI) worsened after doxorubicin (0.38±0.08 vs. 0.41±0.08, p<0.01) and isovolumetric relaxation time (IVRT) showed a significant prolongation after doxorubicin (41.4±1.8 ms vs. 43.1±12.6 ms, p<0.05). Both the tricuspid E velocity (62.3±8.35 cm/s vs. 60.1±7.34 cm/s, p<0.01) and E/A ratio significantly reduced after doxorubicin (1.54±0.26 vs. 1.40±0.23, p<0.01). TDI parameters also showed a significant decrease (tricuspid E’ velocity 15.0±3.37 vs. 14.0±2.84 cm/s, p<0.01).

Left ventricular functions before and after doxorubicin

In this study, the M-mode dimensions of the left ventricle (LV) showed significant changes after doxorubicin except for LVIDs. The LV fractional shortening (LVFS) and ejection fraction (LVEF) both showed reduction as compared to baseline values (32.1±2.26% vs. 31.4±2.27%, p<0.01 and 64.60±4.69% vs. 63.10±4.63%, p<0.01, respectively) (Table 4).

**Table 4:** Conventional echocardiography parameters of left ventricle. EF: ejection fraction; FS: fractional shortening; LVIDd: left ventricular internal diameter in diastole; LVIDs: left ventricular internal diameter in systole

Parameter	Before (n=28)	After (n=28)	p-Value	t-Statistic
EF%	64.6 (4.69)	63.1 (4.63)	<0.01	4.97
FS%	32.1 (2.26)	31.4 (2.27)	<0.01	4.70
LVIDd (mm)	37.8 (6.71)	36.7 (6.76)	0.008	5.86
LVIDs (mm)	24.5 (5.29)	24.3 (5.49)	<0.01	2.56
Mitral E velocity (cm/s)	85.6 (11.3)	83 (11.9)	<0.01	6.26
Mitral A (cm/s)	49.5 (8.41)	52.4 (8.99)	<0.01	-9.16
Mitral E/A	1.78 (0.43)	1.63 (0.39)	<0.01	9.66

Two patients in our study had FS drop below the normal reference range (i.e., 28-38%) post-doxorubicin induction. However, no patients showed a decrease in their FS by 10% of their baseline values. Left ventricular myocardial performance index (LV MPI) worsened after doxorubicin and IVRT prolongation was noticed (0.44±0.05 vs. 0.46±0.06, p<0.01 and 58.6±8.75 ms vs. 60.3±10.1 ms, p<0.05, respectively).

TDI parameters also showed a significant decrease (mitral E’ velocity 15.5±1.96 cm/s vs. 14.7±1.80 cm/s, p<0.01). Mitral E velocity (85.6±11.3 cm/s vs. 83±11.9 cm/s, p<0.01) and E/A ratio decreased after doxorubicin (1.78±0.43 vs. 1.63±0.39, p<0.01) (Table 5).

**Table 5:** Tissue Doppler imaging parameters of left ventricle. LV IVCT: left ventricular isovolumetric contraction time; LV IVRT: left ventricular isovolumetric relaxation time; LV ET: left ventricular ejection time; LV MPI: left ventricular myocardial performance index

Parameter	Before (n=28)	After (n=28)	p-Value	t-Statistic
LV IVCT (ms)	56.8 (9.76)	57.0 (10.1)	0.771	-0.753
LV IVRT (ms)	58.6 (8.75)	60.3 (10.1)	0.999	-3.648
LV ET (ms)	262 (27.9)	251 (27.3)	<0.01	11.36
LV MPI	0.441 (0.05)	0.467 (0.06)	<0.01	-6.49
Mitral E’ velocity (cm/s)	15.5 (1.96)	14.7 (1.80)	<0.01	9.02
Mitral E/E’	5.55 (0.58)	5.66 (0.61)	0.995	-2.80

## Discussion

The mechanism of action of anthracyclines is to act on topoisomerase II, an enzyme responsible for the generation of double-stranded DNA breaks [7]. These drugs bind to and stabilize topoisomerase II-DNA cleavable complexes, resulting in DNA double-strand breaks which are lethal if unrepaired. The mechanism of cardiotoxicity by these drugs is multifactorial but reactive oxygen species (generated due to intracellular metabolism of anthracyclines) induced damage appears to be the predominant mechanism [8]. Other mechanisms that might be responsible for cardiotoxicity are iron accumulation in mitochondria, lipid peroxidation, nitrosylation of proteins, and abnormalities of calcium handling. Whatever the mechanism of toxicity, doxorubicin has the potential to cause both systolic and diastolic dysfunction [9], which can be detected early using echocardiography [10]. In the current study, we utilized conventional echocardiography and tissue Doppler imaging with an aim to determine the acute cardiotoxic effects of doxorubicin in pediatric patients with acute lymphoblastic leukemia after induction dose.

Right ventricular function after induction dose doxorubicin

Right ventricular function assessment by echocardiography is usually not straightforward as several geometrical assumptions are to be made. However, parameters like TAPSE (derived by M-mode), MPI, and velocities (derived by TDI and PW-TDI) do not need any such assumptions and can be used in clinical settings for the assessment of right ventricular function [11]. In this study, TAPSE is significantly decreased post-induction dose of doxorubicin, suggesting RV systolic function is affected after an induction dose of doxorubicin. This is in contrast to the results reported in children after chemotherapy during mid-term follow-up [12]. In our study, we have also seen an obvious change in the diastolic function of RV and this is demonstrated by TDI-derived RV E’ velocity decreasing after induction dose doxorubicin. These findings are similar to the results of the studies done in adult breast cancer patients and pediatric patients with acute hematological malignancies [13,14]. This right ventricular dysfunction could be attributed to increased pulmonary vascular resistance but we could not see any pulmonary regurgitation or tricuspid regurgitation. Moreover, there is no change in Doppler-derived pulmonary acceleration time. Thus, the right ventricular dysfunction observed in our study may be attributed to myocardial injury related to doxorubicin. This is further supported by a change in TDI-derived MPI of the right ventricle.

Left ventricular function after induction dose doxorubicin

In our study, LV developed both systolic and diastolic dysfunction. There is a significant decrease in parameters of systolic dysfunction like LVEF and LVFS. After doxorubicin, LVFS showed a significant reduction in two patients (reduction in their FS to less than 28%), and the rest of the patients had FS in their normal range (though decreased from baseline value). This can be explained by the fact that the LVFS reflects the combined longitudinal, circumferential, and rotational function and all components may not be affected to the same extent after doxorubicin. The LV myocardium has predominantly circumferentially oriented fibers and longitudinally oriented fibers are sparse [15]. The sub-endocardial region is mainly composed of longitudinally oriented fibers, these are susceptible to the deleterious effect of hypoxia in particular [16]. The LV diastolic dysfunction in our study is depicted by mitral inflow Doppler parameters and mitral E/A ratio - both decreased after doxorubicin, similar to several other studies. However, the E/E’ ratio did not change significantly after doxorubicin and this is an end-diastolic pressure parameter of LV. This is contrary to previous studies reported following chemotherapy [14,17,18].

The left ventricular dysfunction observed in our study may be due to DNA damage and free radicle injury of cardiac myocytes by doxorubicin [19]. However, acute leukemia patients have multiple other comorbidities that can affect left ventricular function. More commonly these patients have severe anemia, sepsis, and hyperhydration states which have the potential to affect left ventricular function. Anemia decreases the oxygen content of blood and causes relative myocardial hypoxemia. Blood viscosity is decreased and this in turn leads to increased venous return and preload, decreased afterload, and increased heart rate (through sympathetic stimulation) [20], sepsis can cause septic myocardial dysfunction [21], and hyperhydration can increase the preload [22]. In our study, the median hemoglobin was 8.55 g/dL and this was not low enough to cause significant left ventricular dysfunction. Our study participants were also not in a state of severe sepsis or a hyperhydrated state at the time of recruitment and the systolic and diastolic dysfunction can be attributed to doxorubicin.

Limitations

The main limitation of our study was the cross-sectional nature of the study design. Whether these observed changes are transient or permanent can only be determined through follow-up. Furthermore, biochemical markers of cardiac injury were not measured and were not correlated to echocardiography parameters.

## Conclusions

Acute cardiotoxic effects can occur as early as 72 h after the induction dose of doxorubicin in pediatric acute lymphoblastic leukemia patients. These effects can be in the form of systolic as well as diastolic dysfunction and both ventricles are prone to develop these effects. The clinical signs and symptoms will not be obvious and hence echocardiography (both conventional and TDI) should be used to detect these changes. Multiple comorbidities associated with the disease process can affect ventricular function. Whether these changes are transient or permanent can be known only by follow-up and hence periodic follow-up of these patients is required. Decisions regarding therapeutic dose change or alternative medication can be taken based on serial echocardiography results.
